# Assessing the use of cell phones to monitor health and nutrition interventions: Evidence from rural Guatemala

**DOI:** 10.1371/journal.pone.0240526

**Published:** 2020-11-03

**Authors:** Francisco Ceballos, Manuel Alejandro Hernandez, Francisco Olivet, Cynthia Paz

**Affiliations:** 1 Markets, Trade and Institutions Division, International Food Policy Research Institute (IFPRI), Washington, DC, United States of America; 2 Escuela de Ingeniería en Agronomía, Universidad Mariano Galvez, Ciudad de Guatemala, Guatemala City, Guatemala; Helen Keller International, SIERRA LEONE

## Abstract

In-person (face-to-face) data collection methods offer many advantages but can also be time-consuming and expensive, particularly in areas of difficult access. We take advantage of the increasing mobile phone penetration rate in rural areas to evaluate the feasibility of using cell phones to monitor the provision of key health and nutrition interventions linked to the first 1,000 days of life, a critical period of growth and development. We examine response rates to calendarized text messages (SMS) and phone calls sent to 1,542 households over a period of four months. These households have children under two years old and pregnant women and are located across randomly selected communities in Quiche, Guatemala. We find that the overall (valid) response rate to phone calls is over 5 times higher than to text messages (75.8% versus 14.4%). We also test whether simple SMS reminders improve the timely reception of health services but do not find any effects in this regard. Language, education, and age appear to be major barriers to respond to text messages as opposed to phone calls, and the rate of response is not correlated with a household’s geographic location (accessibility). Moreover, response veracity is high, with an 84–91% match between household responses and administrative records. The costs per monitored intervention are around 1.12 US dollars using text messages and 85 cents making phone calls, with the costs per effective answer showing a starker contrast, at 7.76 and 1.12 US dollars, respectively. Our findings indicate that mobile phone calls can be an effective, low-cost tool to collect reliable information remotely and in real time. In the current context, where in-person contact with households is not possible due to the COVID-19 crisis, phone calls can be a valuable instrument for collecting information, monitoring development interventions, or implementing brief surveys.

## Introduction

Development programs and interventions are often implemented across remote, isolated areas. Regular data collection regarding these interventions is essential to conduct appropriate oversight and monitoring, identify bottlenecks and improve ongoing processes, and evaluate outcomes. While in-person (face-to-face) methods offer many advantages, including increased trust between interviewee and interviewer and the possibility to capture detailed responses in complex contexts, they can be expensive, time-consuming, and logistically challenging, particularly in areas of difficult access. In these situations, mobile phones can offer an alternative approach for collecting ground data that is relatively inexpensive and easily scalable. Moreover, in the current context of travel restrictions, lockdowns, and social distancing measures due to the COVID-19 crisis, the use of information and communications technology (ICT) such as mobile phones may represent the only available method for data collection.

Such a tool for data collection among disadvantaged populations in generally remote areas has only recently become possible at a large scale. Mobile phone penetration has increased dramatically over the past two decades. Global mobile subscriptions have increased three-fold from 34% of the population in 2005 to 108% in 2019 [1]. Current estimates indicate that 66.9% of individuals worldwide own and use a cellphone, though these measures do not take into account indirect access through a family member [2]. Such penetration rates are also true in developing countries and even in isolated rural areas, where mobile penetration is lower than in urban areas but still high and increasing. In Guatemala, for example, the rate of national mobile subscriptions reached 117 per 100 inhabitants in 2018 [3], and it is estimated to be around 90 subscriptions per 100 inhabitants in rural areas.

This paper evaluates the feasibility of monitoring the provision of health and nutrition interventions in rural, remote areas in the department of Quiche, located in the Western Highlands of Guatemala, by directly engaging with end-users through text messages (henceforth SMS) and calls to their mobile phones. The study is part of a broader collaboration with local stakeholders to develop innovative tools to better monitor and improve maternal and child health and nutrition among vulnerable populations in Guatemala. In this line, the study also aims to assess and validate a monitoring tool that can serve to identify bottlenecks in real time and improve the responsiveness of multiple ongoing interventions remotely. The study builds on an earlier proof of concept study conducted in 2017 in the department of Chiquimula, where similar interventions were exclusively monitored by SMS [4]. We expand on this by incorporating phone calls as an alternative communication method while continuing to send SMS to another group of households, allowing for a comparison between both modes of contact. Moreover, the current study takes place in a context with cultural and linguistic challenges, since the department of Quiche comprises predominantly indigenous populations speaking different languages (the areas covered in the present analysis include Ixil, Spanish, Kiche, and Qeqchi speakers). This enables the evaluation of the monitoring tool in a context of linguistic heterogeneity, informing the validity of the instrument in a multi-lingual setting.

To do this, we directly ask households questions about receipt of scheduled health and nutrition services, either through simple SMS or phone calls, over the course of four months. We also send SMS reminders to a subgroup of households to assess the effects on attending health centers to receive these services. We rely on a cluster randomized controlled trial (cRCT) design to compare the relative effectiveness, as measured by response rates, between these two modes of contact, with and without the provision of reminders. We examine the potential factors correlated with the likelihood of responding, including language and location differences. In addition, we compare direct answers from households with administrative data maintained by the health centers for a subsample of our study group.

Monitoring these interventions is highly relevant in both a country and area where food insecurity and chronic malnutrition is a serious problem. Despite its level of Gross Domestic Product (GDP), Guatemala is the Latin American country with the highest chronic malnutrition rate for children aged 0–5 years old (46.5%) and is ranked sixth globally [5, 6]. The department of Quiche has, in turn, a chronic malnutrition rate of over 68%, and was one of the departments prioritized by the Government of Guatemala under the 2016–2019 National Strategy for the Prevention of Chronic Malnutrition (ENPDC) to provide several health, nutrition, and complementary services. The ENPDC was preceded by the 2012–2015 Zero Hunger Pact (PPH0), which also prioritized several municipalities from Quiche [7–9]. We specifically focus on monitoring key health and nutrition interventions linked to the first 1,000 days of life, from conception to two years of age, a critical window of growth and development [10–12], commonly referred to as the 1,000 Days Window of Opportunity.

The use of ICT on issues related to poverty, health, and nutrition is not new in the literature. One strand of studies has concentrated on evaluating the use of ICT to provide information on prices, pests, diseases, good hygiene practices, and nutrition reminders or warnings to aid better decision-making or promote behavioral changes among the population of interest [13–19]. Another strand of the literature has focused on assessing the use of ICT for data collection purposes. A study in Scotland tested the validity and practicability of text messaging for collecting information on infant feeding methods and future feeding plans and found it to be a reliable method [20]. Three studies in rural China compared text messaging versus face-to-face interviews for brief child health and nutrition surveys and found low to moderate SMS response rates but high agreement with face-to-face responses [21–23]. A study in Denmark also found a high concordance between SMS responses and retrospective telephone interviews among low back pain patients [24], while a recent study in Malawi showed that mobile-phone interviews could offer a low-cost alternative to assess the strength of implementation of a family planning program [25].

We contribute to both strands of the literature by comparing the benefits of two alternative modes of communication (SMS and phone calls) within the same context using experimental methods. We also evaluate the effects of providing SMS reminders to encourage the reception of health and nutrition services among children and pregnant women in remote, rural areas. Similarly, we inform the robustness of such data collection tools in a context of rugged geography and linguistic heterogeneity and offer preliminary evidence on the accuracy and veracity of direct household responses by comparing these with administrative data.

## Materials and methods

The study assesses the use of cell phones to monitor health and nutrition interventions related to the 1,000 Days Window of Opportunity, which are part of the core interventions of the national strategy against malnutrition (ENPDC) in Guatemala. As such, the study focuses on pregnant women and children under two years old and the key health and nutrition interventions they are normally meant to receive from their local public health centers.

Participation in the study was strictly voluntary and all participating households gave written, informed consent. The study was approved by the Institutional Review Board of the International Food Policy Research Institute (IFPRI).

### Study design

The study was carried out between November 2018 and June 2019. The study design incorporates lessons learned from a proof of concept study implemented in the department of Chiquimula in 2017. The most important lesson drawn from that study was the difficulty of asking questions over SMS, bringing about the possibility of communicating via phone calls instead, due to (i) a greater ease by households to answer phone calls, (ii) the ability to resolve doubts about the questions asked on the spot during a call, and (iii) the null cost of answering incoming calls in Guatemala. We accordingly decided to incorporate phone calls in the present study.

Two other major conclusions from the previous study were that financial incentives (in the form of airtime top-ups provided if a household answered all questions asked) and the number of questions sent (to assess potential saturation effects) did not turn out to be determining factors in the propensity of households to respond. We thus chose not to reassess these factors in Quiche. Instead, all participating households received the entire set of questions around the health and nutrition interventions that were due, and households that were asked questions through SMS also received monthly airtime top-ups (to avoid liquidity constraints preventing households from responding). Other lessons learned from the proof of concept study were to communicate during weekends (since shared use of a mobile phone within the family implied a lack of access during weekdays) and to exclusively rely on women for contacting households (due to issues with unknown men calling women’s cell phones).

Finally, following the behavioral change literature using ICTs, an additional purpose of this study was to assess the effects of sending reminders via SMS to encourage individuals to visit the health center to receive interventions. Thus, SMS reminders were sent about a week in advance of the date in which the targeted individual was due to receive a given health or nutrition service according to the official health protocol. S1 Table provides further details of the study elements included in Quiche versus Chiquimula.

Based on these study objectives, we implemented a clustered randomized controlled trial (cRCT), randomly assigning every participating community to one out of four treatment arms, with all households in the community subject to the same treatment. We followed a randomized crossover design along the following dimensions:

- Mode of contact. Households in half of the selected communities would be asked questions through SMS, while those in the other half of communities would be asked questions through phone calls.
- Reminders. In addition to the monitoring questions, households in half of the selected communities would be sent SMS reminders for all upcoming health services for the targeted individual during the course of the study and those in the other half would receive just the monitoring questions (by phone or text) but no SMS reminders.

This design has the dual advantage of both reducing the possibility of spillovers between treatment arms and simplifying field logistics. The randomization of communities was carried out before fieldwork, such that the field team doing the interviews would know in advance the treatment arm to which a community belonged to and could fully explain to participating households what to expect over the subsequent weeks.

### Sample size

The department of Quiche was selected since it was one of the four departments prioritized in the ENPDC and because Mayan languages are spoken across the large majority of its communities, allowing for the assessment of potential linguistic obstacles. In consultation with local actors, the municipalities of Santa Maria Nebaj (hereafter Nebaj) and San Miguel Uspantan (hereafter Uspantan) were selected taking into consideration: (i) the availability of health services, such that sufficient coverage of health services would exist in the selected municipalities to allow for a monitoring scheme; and (ii) the absence of social conflicts, to avoid potential disruptions to fieldwork activities.

Due to budget limitations that precluded the possibility of conducting fieldwork across all communities within each municipality, close to 50% of the communities in both municipalities were randomly selected for the study, stratifying by local language, access to electricity, and population. Naturally, communities with no cell phone coverage, as informed by local authorities, were excluded from the sampling frame (representing around 15% of the communities in each municipality).

An initial (brief) in-person household listing exercise was conducted in each selected community between November 30 and December 23, 2018. Field staff visited every dwelling in the community, interviewed the household living in it in order to identify those households with targeted individuals (i.e. households with children under two years old and pregnant women), and collected their phone numbers and other basic information. Across all surveyed communities, a total of 2,559 households had at least one child under two years old or one pregnant woman (if the household had more than one member from these demographic groups, a single member was selected within each household and is hereafter referred to as the targeted individual). Of these households, 2,374 (93%) also had access to a cellphone and at least one literate member, which were additional requisites for eligibility. Since the health and nutrition interventions that the targeted individual should receive depend on the calendar established by the official health protocol, individuals from some eligible households did not have an intervention due in the relatively short timeframe of the study’s monitoring period (four months). As a result, only 1,542 of the above households were ultimately eligible for monitoring, which started the last week of January 2019. Refer to S1 Appendix and S2 Table for additional details on the household listing and the selection of the monitored sample.

**Fig 1:**
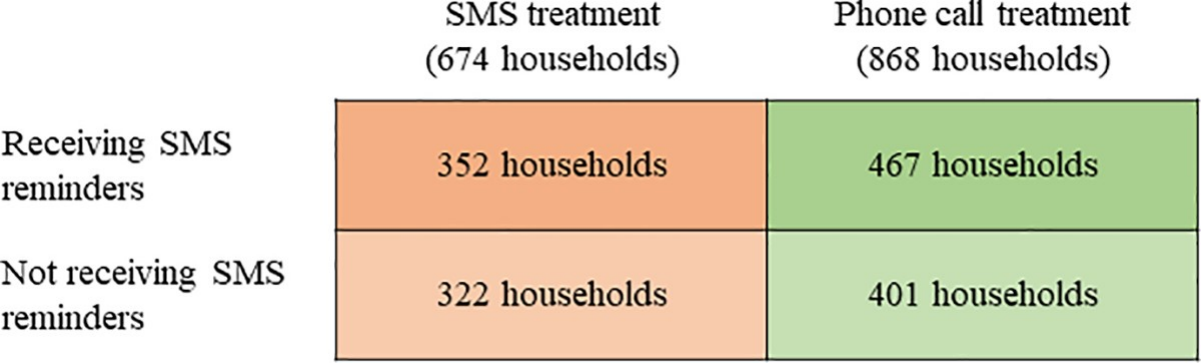
Treatment arms arising from the randomized crossover design at the community level.

Fig 1 depicts the final distribution of the 1,542 households by treatment arm. The random assignment did not result in all groups being of identical size, as the size of communities varies over time and was not known in advance. S3 Table shows balance across the phone calls and SMS treatment arms for a host of household characteristics as a result of the randomization. On average, both groups have similar socioeconomic characteristics, are located at a similar distance to the nearest health center, and had similar health and nutrition interventions due.

### Monitored interventions

The Ministry of Health in Guatemala (MSPAS) produces guidelines defining a series of health and nutrition services ought to be administered at official health centers to pregnant women and children under two years old, at specific months of age (in the case of children) or months of gestation (in the case of pregnant women). Since some health and nutrition services indicated in these guidelines were facing supply constraints and were not being administered at the time of the study, we decided to exclude these, resulting in 13 services for direct monitoring. Table 1 details these services or interventions, where interventions 1 through 10 are for children under two years old and interventions 11 through 13 are for pregnant women.

**Table 1:** Interventions monitored during the study.

#	Target	Intervention	Activity
1	**Children under 2 years old**	**Exclusive Breastfeeding**	Counseling for exclusive breastfeeding during the first six months of life
2		**Vitamin A**	Provision of vitamin A supplements
3		**Powdered Micronutrients**	Provision of 1 packet, once a day, over 60 days
4		**Vaccines**	BCG (Bacilo Callmette Guerin)
5			OPV (Oral Polio Vaccine, 3 doses)
6			Pentavalent (DPT-HBV-HIB, 3 doses)
7			MMR (Measles, Mumps, and Rubella)
8			OPV 10
9			DPT 10
10			Rotavirus (2 doses)
11	**Pregnant women**	**Care during Pregnancy**	Provision of 12 folic acid 5mg pills
12			Provision of 24 ferrous sulphate 300mg pills
13			4 control visits throughout pregnancy

During the household listing in the selected communities, the age (in months) of children and the months of gestation of pregnant women were collected. Using this information, we were able to determine all upcoming health services that a given targeted individual was due to receive during the monitoring period, resulting in a household-specific calendar of questions to monitor the receipt of these services. Hence, the total number of questions asked varied across each targeted individual. For example, a household with a 2-month-old child at the time of the listing received a total of seven questions during the four months of the effective monitoring period, while a household with a 7-month pregnant woman received three questions in the same period. S2 Fig details the number of monitored interventions (questions asked) by age of the child and months of pregnancy.

Questions were administered (either by SMS or phone call) one month after the child or pregnant woman was due to receive each health service. As an example, the first dose of the Pentavalent vaccine should be administered at two months of age, so the question was asked when the child turned three months. This one-month window allowed sufficient time for the targeted individual to visit the health center, while at the same controlling for the adequate timing of the service provision according to the MSPAS protocol. The calendar also served to send SMS reminders among the treatment group that was assigned to receive reminders, about a week in advance to the date in which the child or pregnant woman was due to receive each health service.

Since the monitoring questions (and reminders) rely on the official guidelines for the timing of services and not on actual appointments made by households with their respective health centers, we also avoid any potential selection bias that may result from differences between households securing and not securing appointments in advance. As the age of the child or month of pregnancy are the only variables determining the interventions to be received and monitored (following the guidelines), and these are ultimately balanced across treatment arms, we do not believe selection bias to be a concern in this regard.

### Data collection method

**Fig 2:**
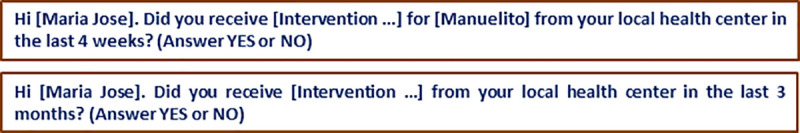
Structure of monitoring questions.

Fig 2 depicts the general structure of the two types of questions asked, depending on the nature of the intervention being monitored, across both SMS and phone calls (see S1 Fig for the full set of questions asked for all monitored interventions). To make the questions approachable, these were written in a colloquial language and carried the first name of the contact person (pregnant woman or mother of the child) and that of the child, when applicable, using the information (names) obtained during the listing. The questions asked had to be simple and direct in order to avoid difficulties in understanding and maximize response rates, considering the relatively low level of education in the study area. Similarly, the messages had to be concise due to the SMS 164-character limit. To maximize response accuracy, participating households were also instructed (during the listing) to consult their individual-specific health card prior to responding to a SMS or phone call. This card is provided and updated by health workers every time someone visits a health center.

While phone calls were made in the main language spoken in the community where the household was located, SMS communication was solely in Spanish for two main reasons. First, local authorities indicated that households tend to use Spanish for reading and writing, even when the local language predominates orally. Second, for compatibility purposes with the automated SMS delivery system, the text messages could not contain certain characters commonly used in the written forms of local Mayan languages. Questions asked over SMS further made explicit that a “YES” or “NO” answer was required (which was also explained during the households’ visits), to simplify both the answering process for households and the processing of responses.

Since asking whether a child or pregnant woman has received a service they are supposed to receive (based on the official guidelines and their age or months of pregnancy) poses minimal concerns around confidentiality, we did not explore the feasibility or costs associated with sending encrypted SMS messages. This is an important aspect that should be taken into account when monitoring other type of interventions such as those related to individual-specific health issues, where protecting the confidentiality of participants may be a concern.

### Monitoring implementation

As noted above, all eligible households were fully informed of the study during the initial, in-person household listing (where phone numbers were collected). A brochure was also provided with the details of the study to all participating households in their local language. Prior to the start of the monitoring period, we further advertised the study through brief interviews in the local radio and television (both in Spanish and in the local language) in order to both remind participating households about the study and encourage them to answer the questions they would be receiving during the upcoming weeks. Similarly, we participated in meetings with local authorities from the communities in both municipalities to communicate the objectives and scope of the study and request their support over the following weeks.

The monitoring implementation began roughly one month after the initial household listing with a welcome message to all participating households indicating that they would be receiving questions either by SMS or phone calls (depending on the treatment group) in the upcoming months asking about the health and nutrition services received by the targeted child under two years of age or the pregnant woman. The households in the SMS treatment group also received an initial airtime top-up of 5.5 quetzales (approximately 0.75 US dollars) and an additional SMS informing that these top-ups were provided to help them to answer the questions they would be receiving in the following weeks. The first batch of questions was made on January 27 and the last one on May 26, 2019.

SMS-based questions were sent using an automated system for sending and receiving SMSs from a local company. In the system, a processing rule was created to recognize an answer of “YES” or “NO” in all its variants (e.g., “yes”, “Yes”, “YES”) and to send an automatic response in case the answer did not match this format: “Please answer YES or NO only, we cannot process other responses. Thank you for your participation.” Still, a very small number of responses had to be manually verified and classified as “YES” or “NO.”

Eight surveyors (teleoperators) were hired to make the phone calls: three of them speaking Ixil, two Spanish, one Kiche, and two Qeqchi. The proportion of teleoperators in each language was correlated with the language distribution of the monitored households. Regarding the back-end system for phone calls, each surveyor was provided with an automated Excel spreadsheet with a full list of households to be contacted on a particular date, their contact information, and additional information on household members and the listing interview date so contacted households would trust that the call was associated to the study they had accepted to participate in. The spreadsheet included a dedicated solution in Visual Basic that showed the complete list of questions to be asked to a given household on a given date and an entry form to facilitate the recording of answers. The spreadsheets were saved automatically after each call and shared weekly with the researchers.

The monitoring occurred on Sundays as it is the day in which the majority of household members are at home. Questions sent via SMS were scheduled to be sent on Sundays at noon and the protocol was to wait until the following Saturday for a response, although 82% of the households that responded to SMS did so the same Sunday or during the following day. In the rare cases where more than one SMS response was received over the week, the last message received was considered as the final response. Phone calls were made on Sundays at different times over the day. When a household could not be reached by phone, no voice-mails were left but additional phone call attempts were made within the same day and the following days (mainly between two and five additional attempts in total). On this regard, it is worth acknowledging that phone calls permitted to make several attempts as opposed to SMS that were only sent once; yet the communication (question) sent through SMS is fully transmitted in the first attempt while the communication through phone calls ultimately depends on the household answering the phone (as voice messages were also not left given that not all participants necessarily use this service). In any case, similar to SMS, close to 80% of the households that responded the phone calls did so within the same Sunday or over the following day, and one third did so at the first attempt.

In some cases, the health services’ protocol from MSPAS indicates the provision of more than one service at a specific time during pregnancy or over the first two years of life. For instance, several vaccine doses should be administered at six months of age, during the same visit. In the case of phone calls, all questions could be asked at the same time once communication with the household was established. An SMS-based mode of communication, however, has the disadvantage of not being able to ask more than one question at a given time: if two questions were sent by SMS on the same day and two answers were received, it would not always be possible to accurately match the answers with the corresponding questions without relying on strong assumptions. This is true when the answers differ or when the number of answers received are less (or more) than the number of questions sent. To solve this issue, SMS questions had to be sent one at a time each week in these situations, which also implied that the SMS monitoring period ended four weeks after the monitoring period using phone calls (as noted in the next Section). This limitation is an important constraint that should be taken into consideration in more complex SMS-based monitoring designs.

### Administrative information

In order to compare household responses with the official information that the targeted individual had received, administrative records from specific health centers in the municipality of Nebaj were collected after the completion of the monitoring activities. This information was obtained from the Health Management Information System (SIGSA) maintained by MSPAS, which keeps records of several services provided by health centers serving the population of interest. The frequency in which the system is updated varies significantly across locations, reason why we collected this information about one month after finalized the monitoring.

We focus on vaccines as the administrative records for vaccines received by children under two years old contain detailed information on the name of the child, date of birth, and the mother’s name (besides the household location and date of service), which permits an accurate one-to-one matching with our dataset. Available administrative records for other services or for pregnant women, for instance, do not include birth date, which difficult a precise matching. This additional exercise allows us to evaluate the veracity of the answers provided using a subsample of monitored children in Nebaj as an example.

### Statistical analyses

The data analyses performed in the study are divided into five parts. Given the significant difference in response rates obtained through SMS and phone calls, the analysis for each mode of contact is conducted separately.

First, we report overall response rates obtained throughout the duration of the study and disaggregated over time. This includes aggregate tabulations as well as disaggregation by geographic location, predominant language in the community, and type of intervention.

Second, we examine the association between sending SMS reminders and receiving health services. We compare the shares of households reporting to have received health services, between communities that received and did not receive reminders.

Third, we implement a multivariate regression analysis to formally examine the factors correlated with the likelihood of responding. We estimate the following linear probability model via ordinary least squares (although the results are robust to implementing a discrete choice model),

$$R_{ij}=\alpha +X_{ij}\beta +T_{ij}\delta +c_{j}+u_{ij}$$ (1)

where the dependent variable *R_ij_* is a dichotomous variable that takes a value of one if household *i* in location *j* provided a valid answer and zero otherwise; *X_ij_* is a vector of household characteristics such as gender, age, language, and level of education of household head, household size, and distance to the closest health center; *T_ij_* represents two indicator variables for the type of intervention monitored (if care intervention during pregnancy and if vaccine intervention); *c_j_* is a location fixed effects component (i.e. a municipality or community indicator where the household is located) that controls for unobserved factors at the geographic location level that could likely affect households’ propensity to respond; and *u_ij_* is an idiosyncratic error term. The standard errors in Eq (1) are further clustered by community to account for any correlation in responses within locations. The parameters of interest are *β* and *δ*, which capture the partial correlations of this wide set of potentially-relevant factors on the likelihood of responding. This regression analysis was also complemented with a qualitative analysis through focus groups in selected participating communities.

Fourth, we evaluate the accuracy of household responses by directly comparing the “YES” and “NO” responses received with the information recorded in the health centers’ administrative data. We work with a subsample of households and focus on vaccines as an example, since this permits for an accurate comparison between the two sources of information, as discussed above.

Finally, we discuss the cost-effectiveness of the monitoring tool. We calculate and compare the costs per question sent and per effective response received for both SMS and phone calls.

## Results and discussion

Table 2 summarizes the characteristics of the 1,542 monitored households located in the municipalities of Nebaj and Uspantan. As observed, the vast majority of households participating in the study have a male household head with no or elementary education, and close to 90% mainly speak a language other than Spanish. The average household has more than six members. These demographic characteristics generally resemble a representative rural household in the Western Highlands of Guatemala [6, 26, 27]. Note that the average reported time to get to the closest health center is 21 minutes, and 75% of the households had a child under two years old that was monitored while the remaining 25% a pregnant woman.

**Table 2:** Characteristics of the households participating in the study.

	Mean	Standard Deviation	Min	Max
If Household Head is male	0.87	0.332	0	1
Household Head age	39.13	13.411	16	86
If Household Head speaks non-Spanish language	0.88	0.321	0	1
If Household Head has no education	0.37	0.482	0	1
If Household Head has elementary education	0.41	0.492	0	1
If Household Head has secondary education	0.22	0.412	0	1
Number of household members	6.22	2.708	2	19
Distance to the health center (in minutes)	21.13	26.344	0	240
If pregnant woman monitored	0.25	0.431	0	1
If child under two years old monitored	0.75	0.431	0	1
If household located in Nebaj	0.45	0.498	0	1
Observations				1,542

### Response rates

Table 3 reports the total numbers of questions sent and response received for SMS (Panel A) and phone calls (Panel B). In the case of SMS, a total of 2,970 questions were sent over the four months of the monitoring period and only 427 were answered appropriately (either with a “YES” or “NO”), resulting in a global (valid) response rate of 14.4%. Among the remaining questions sent, most of them were unanswered (2,465 or 83% of the total questions sent), while 78 responses (2.6%) were categorized as invalid since these were not related to the question asked. As a point of reference, a related study in rural China found an SMS response rate of 48.7% [21]. Since the phone numbers provided by households during the listing were verified by the surveyors (by calling the phone number), we infer that most of the questions sent by SMS were received but simply unanswered.

**Table 3:** Questions sent and responses received by SMS and phone calls.

Panel A: SMS			Panel B: Phone calls		
Category	Quantity	Percentage	Category	Quantity	Percentage
Questions sent	2,970	100.0%	Questions sent	3,824	100.0%
Valid responses	427	14.4%	Valid responses	2,900	75.8%
No	82	2.8%	No	598	15.6%
Yes	345	11.6%	Yes	2,302	60.2%
Unanswered	2,465	83.0%	Phone always off	432	11.3%
Invalid answers	78	2.6%	Unanswered	367	9.6%
			Wrong phone number	110	2.9%
			Rejections	15	0.4%

In the case of phone calls, a total of 3,824 questions were asked and 2,900 were answered appropriately, resulting in a global response rate of 75.8%. The larger number of questions asked by phone, compared to SMS, is due to the higher number of households in the communities that were randomly selected to be contacted by phone (see Fig 1). Among the remaining questions, in 432 cases (11.3% of total) the question could not be asked as the phone appeared to be off every time the household was called (perhaps due to the weak mobile signal in some areas); in 367 cases (9.6%) the calls were never answered; in 110 cases (2.9%) the registered phone ended up being a wrong number or belonged to a person who was not aware of the study; and in 15 cases (0.4%) the household answered the call but refused to provide an answer. Overall, the aggregate response rate to phone calls was over five times larger than the response rate to SMS.

The contrast between SMS and phone calls is also evident when observing the inter-household variability in response rates. As shown in S3 Fig, only 13 households that received questions through SMS (about 2% of the total number of households in this treatment group) answered all the questions that were sent to them versus 642 (74%) in the case of households that received phone calls. On the other hand, 426 (63%) households monitored through SMS did not respond to any message sent versus 182 (21%) among the households monitored by phone. It follows that households responding to one question over the phone, are highly likely to respond to all subsequent questions (calls) during the following weeks; in the case of text messages, it is highly likely that households will not respond to any SMS.

**Fig 3:**
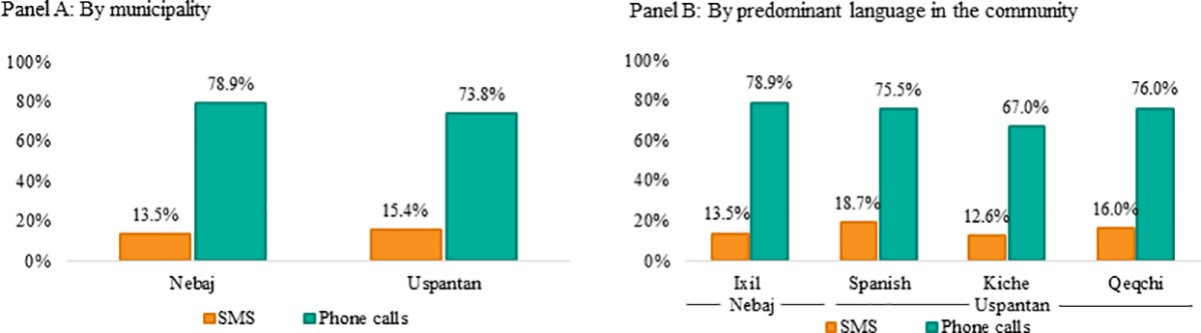
Response rate by municipality and predominant language in the community.

Fig 3 disaggregates the response rates by municipality in Panel A and by main language spoken in the community in Panel B. We observe that the differences in the response rates between SMS and phone calls are very similar between Nebaj and Uspantan. Roughly, of every ten questions made, 7–8 are answered in the case of phone calls, while around 1.5 are answered in the case of SMS across both municipalities. When further disaggregating by the predominant language in the community, the differences between SMS and phone calls generally hold across languages. Recall that phone calls were made in the local language and the response rates in locations where Ixil, Spanish, and Qeqchi predominate are over 75%, while the response rate in locations where Kiche predominates is 67%. Communication by SMS was exclusively in Spanish and in this case the highest response rate is among communities where precisely the predominant language is Spanish (18.7%), while the lowest response rate is among communities where the predominant language is Kiche (12.6%).

**Fig 4:**
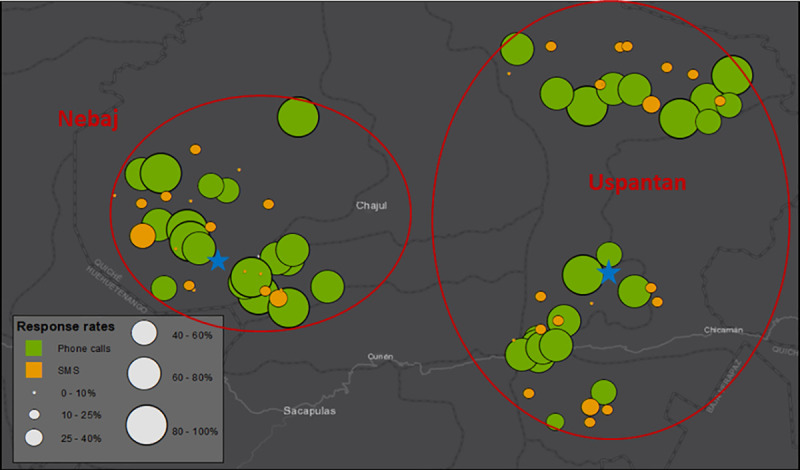
Response rate by community. The stars indicate the location of the main town in each municipality.

Fig 4 maps, in turn, the aggregate response rates across the monitored communities in Nebaj and Uspantan. The size of the circles reflects the share of valid responses in each community. There are no clear spatial patterns between the response rates for both SMS and phone calls and the geographic location of the community. Communities located farther away from the main town in the municipality (such as the Northeastern area of Nebaj and the Northern area of Uspantan) seem equally likely to respond than communities located closer to the main town, which are generally more accessible. As a reference, traveling by car from the main town in Uspantan to the Northern area can take more than five hours given the rugged geography and the very poor road conditions in the area. This suggests that phone calls can be useful for monitoring purposes even in remote, isolated areas.

**Fig 5:**
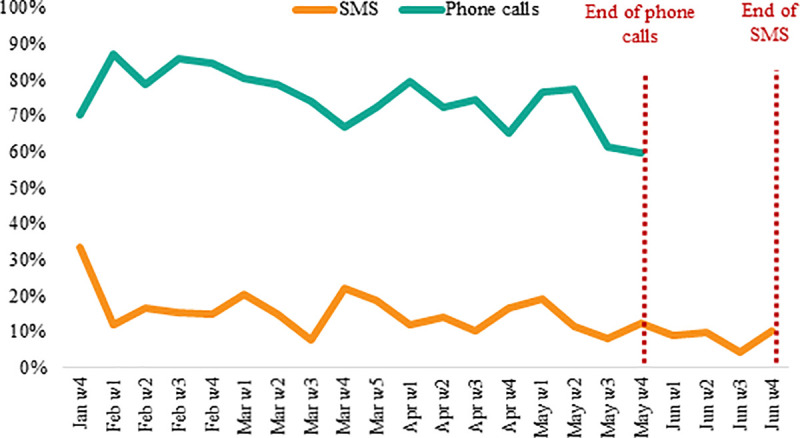
Response rate over time.

We further examine whether response rates changed over time. Fig 5 shows the weekly evolution of the response rate by SMS and phone calls, where SMS responses were collected until late June given that only one question could be asked per week by text as opposed to phone calls (as noted above). In both cases we observe a modest decline in the response rate over time. During the initial batches of questions, the response rate to phone calls fluctuated between 80–85% during the initial weeks and decreased to 65–70% over the last weeks. In the case of SMS, the response rate decreased from 15–20% during the first weeks to around 10% towards the end of the study. In the pilot study in Chiquimula, we also observed a decline in the response to SMS, from 25–30% to 10% over a period of six months.

Lastly, S4 Fig reports the response rates obtained for each of the 13 interventions monitored. Overall, we do not find major differences across interventions both for SMS and phone calls. Except for breastfeeding counseling and BCG vaccine where very few cases (less than five) were monitored, in the case of SMS the response rates fluctuate between 11.2% for prenatal checkups and 17% for rotavirus vaccine, while in the case of phone calls the response rates fluctuate from 74.8% for pentavalent and OPV vaccines and powdered micronutrients to 77.5% for rotavirus vaccine. Overall, response rates do not appear to vary significantly by: (1) targeted individual (i.e. between questions directed at children under two years of age and at pregnant women); or (2) type of intervention (i.e. between vaccines and other services).

### SMS reminders

**Fig 6:**
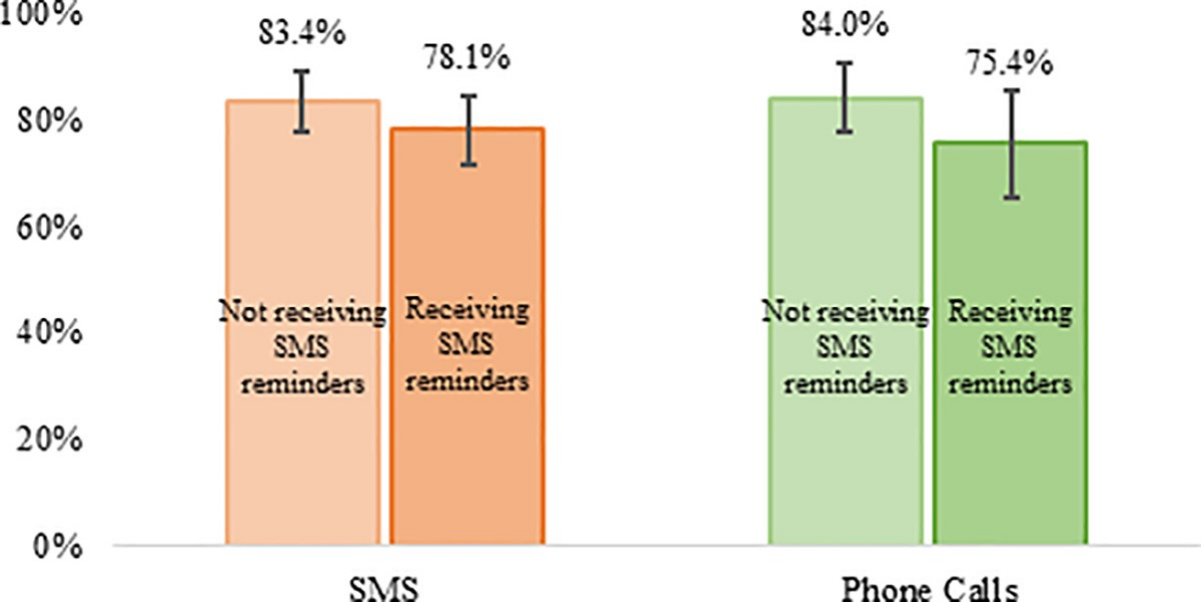
SMS Reminders and reception rate of interventions. The vertical lines correspond to the 95% confidence bands.

Another goal of the study was to evaluate whether sending SMS reminders to households contributed to improving attendance to health centers to receive the corresponding health and nutrition interventions. To test this, we compare the reception rate of services between households in half of the monitored communities that received calendarized SMS reminders and households in the other half of communities that did not receive these. Fig 6 shows that the share of households reporting to have received the scheduled services (over the total number of households reporting valid answers) is higher among communities that did not receive SMS reminders, both when considering communities monitored by SMS and phone calls. These differences, however, are not statistically significant at conventional levels in either case.

Hence, sending SMS reminders does not seem to be positively associated with a higher reception of health and nutrition interventions. This finding contrasts with other studies [13–17], which show evidence for the use of text messaging as a tool for behavior change. Two factors can explain this differing finding. First, the interventions included in this study are basic health and nutrition services that children and pregnant women are supposed to receive on their regular (scheduled) visits to health centers following the standard official protocol, such that sending a reminder may not have an additional effect on the probability of attending and/or receiving the intervention. Second, communication via SMS does not appear to be an effective mode of contact with households in Guatemala (both studies in Chiquimula and Quiche confirm this). The focus groups conducted at the end of the study suggests that sending reminders via phone calls could be more effective than sending SMS, which is an aspect to be tested in future studies (see S2 Appendix).

### Factors correlated with the likelihood of responding

This subsection discusses the key identified factors associated with the likelihood or probability of responding based on the multivariate regression analysis defined in Eq (1). S4 and S5 Tables show the full estimation results for each mode of contact. Column (1) in each table controls for the municipality where a household resides while column (2) controls for the community in which a household is located. For ease of presentation, we focus on column (2) results (i.e. the most comprehensive model).

**Fig 7:**
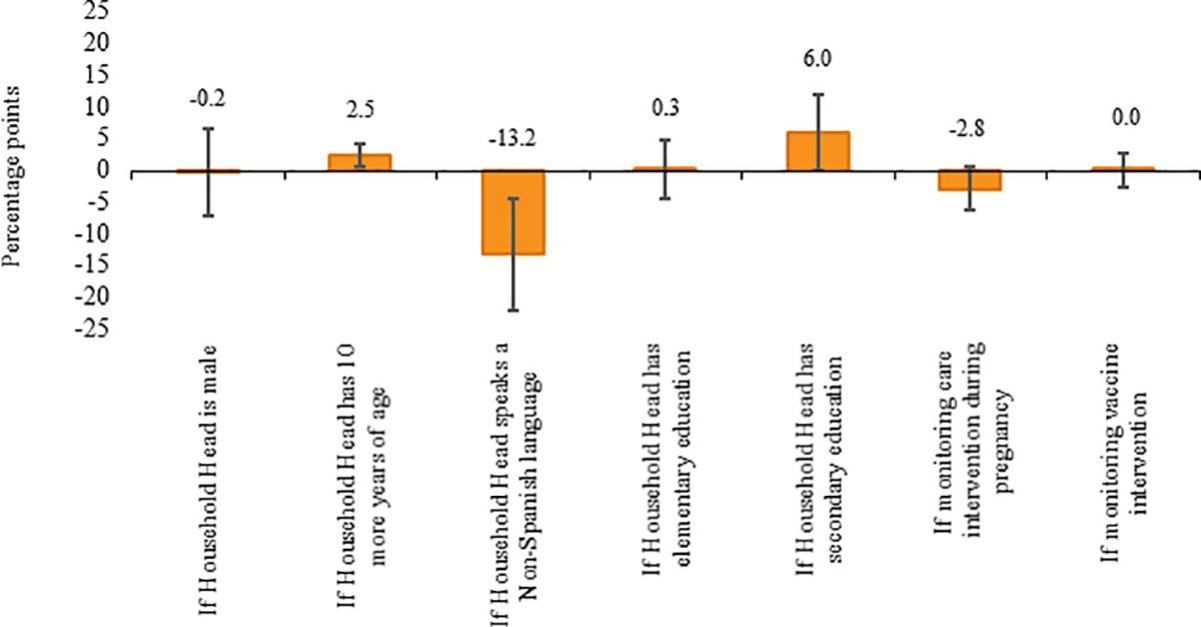
Marginal effects of selected variables on the probability to respond via SMS. The vertical lines correspond to the 95% confidence bands.

Fig 7 presents the corresponding partial correlations (marginal effects) of selected variables on the likelihood of responding via SMS. In line with the findings discussed above, the probability of response does not vary according to whether the questions refer to interventions aimed at pregnant women or at children under two year of age, or whether they refer to vaccines versus other interventions. While the gender of the household head also does not seem to matter, the language spoken in the community, and the education and age of the household head are correlated with the probability of responding to SMS. If the main spoken language of the household head is a non-Spanish (Mayan) language, the probability of responding to an SMS (sent in Spanish) is 13.2 percentage points lower than if the main spoken language were Spanish. Likewise, if the household head has completed secondary or higher education, the probability of response increases by six percentage points (compared to those households where the head does not have any education). The probability of response is also slightly higher among households with an older head (ten more years of age is associated with a 2.5 percentage points increase in the likelihood of response).

**Fig 8:**
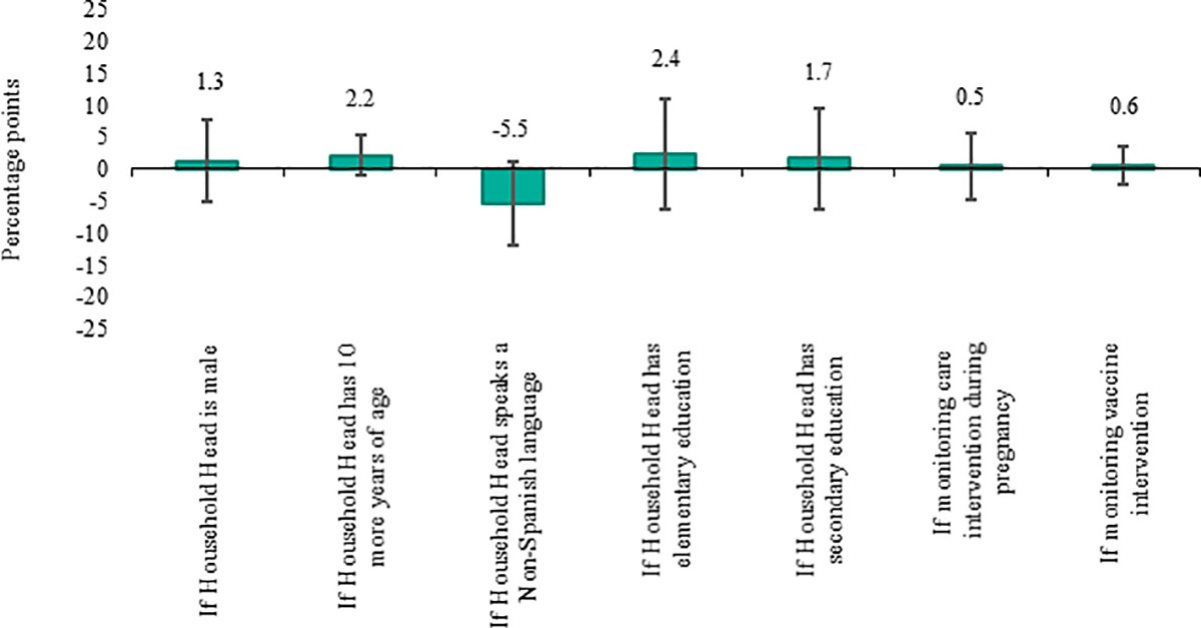
Marginal effects of selected variables on the probability to respond via phone calls. The vertical lines correspond to the 95% confidence bands.

Fig 8 reports the marginal effects of the selected variables on the likelihood to respond via phone calls. In this case, none of the modeled potential factors are correlated with the probability to respond. As opposed to SMS, language, education, and age do not appear to significantly affect the likelihood of providing valid responses through a phone call. Similarly, the likelihood of responding a phone call is not affected by whether the household head is male or female, whether the monitored interventions are aimed at pregnant women or at children under two years old, or whether the monitored intervention refers to a vaccine or other health services.

The quantitative analysis was complemented with a qualitative assessment based on four focus groups we conducted after concluding the monitoring period study. Two communities were selected in Nebaj (one that received SMS and one that received phone calls) and two additional communities in Uspantan. Among participants that received SMS, the main reasons mentioned for not answering questions were a lack of airtime (despite the airtime top-ups provided by the project) and not having been notified about the SMS (as cell phones are usually shared between several household members). Among participants that received phone calls, the few that did not respond also mentioned that they were not notified about the call, as well as other reasons such as a lack of phone access during the day, lack of understanding of the question, or that they had lost their phone. Refer to S2 Appendix and S6 Table for further details. Another important finding was that all participants, regardless of whether they received SMS or phone calls, expressed their strong preference for communicating through phone calls.

### Match between household responses and administrative records

A final piece to assess the viability of using cell phones to monitor health and nutrition interventions is to evaluate the veracity and accuracy of responses provided directly by households. For this, we examine whether household responses match with the information recorded in the Health Management Information System (SIGSA) maintained by MSPAS, which keeps official records of the different services received by every individual visiting the public health centers. In the case of children under two years old, the administrative records contain detailed information of the different vaccines administered together with the date of service, which can be directly compared with the responses by household. Ultimately, we do not only want to determine whether households respond or not (in real time) and their preferred mode of contact, but also whether they respond correctly or not.

Table 4 presents the percentage of agreements (i.e. “Received”/“Given” and “Not received”/“Not given”) and disagreements (i.e. “Received”/“Not given” and “Not received”/“Given”) between household responses and the official records for selected vaccine interventions for a subsample of 280 children under two years old located across monitored communities in Nebaj. Overall, there is an 84% match between households’ SMS responses and administrative records and a 91% match between household responses through phone calls and administrative records. When disaggregating these by specific vaccine, the rates fluctuate between 63% and 96% among SMS responses, and 88% and 95% among phone call responses. We take this as evidence that the responses received are in most cases accurate despite the mode of contact: even though the response rate by SMS is low the response accuracy is high, while in the case of phone calls response rates are both high and accurate. Certainly, the few observed discrepancies may not only be due to household error but could also indicate errors on the administrative records.

**Table 4:** Match between household responses and administrative (SIGSA) records in Nebaj for selected vaccine interventions.

Household responses	SIGSA records			
	SMS		Phone calls	
	Not given	Given	Not given	Given
Pentavalent Vaccine				
Not received	0%	5%	1%	6%
Received	5%	90%	6%	87%
OPV Vaccine				
Not received	0%	0%	1%	4%
Received	10%	90%	6%	89%
MMR Vaccine				
Not received	0%	4%	1%	2%
Received	0%	96%	5%	92%
OPV-10 Vaccine				
Not received	0%	8%	0%	7%
Received	8%	84%	5%	88%
Rotavirus Vaccine				
Not received	0%	13%	2%	2%
Received	7%	80%	5%	91%
DPT-10 Vaccine				
Not received	0%	37%	0%	0%
Received	0%	63%	5%	95%
Total				
Not received	0%	11%	1%	4%
Received	5%	84%	5%	90%

Note: Information based on 280 monitored children in Nebaj that were successfully matched with the SIGSA records.

### Cost-effectiveness of responses

Lastly, we discuss the overall cost-effectiveness of monitoring interventions through cell phones in our study, which helps put our results in perspective for eventual replications or scaling up of a monitoring system of this nature. It is worth noting that over 75% of the total fieldwork budget was devoted to the initial household listing in the selected communities in order to identify households with targeted individuals and collect their phone numbers and other necessary information. These costs could surely be avoided if household records, including telephone numbers, were collected and maintained by the corresponding local administrative and health authorities, such that households could be contacted without previously visiting them. The remaining share of the budget (less than 25%) was devoted to the monitoring itself.

As shown in Table 5, communicating with households through SMS is actually slightly more expensive than communicating through phone calls. In particular, sending questions through SMS requires the use of a specialized platform in addition to the provision of airtime top-ups to households, at a total cost of approximately 3,314 US dollars, or 1.12 dollars per message sent. Phone calls required hiring eight (part-time) teleoperators, as well as the provision of monthly pre-paid plans for unlimited telephone calls to each operator, at a total cost of approximately 3,253 dollars or 0.85 dollars per call. However, if we further take into account the large difference in response rates under both modes of communication, the cost per effective response is dramatically lower in the case of monitoring by phone calls compared to that by SMS (1.12 dollars versus 7.76 dollars, respectively). We conclude that establishing a part-time call center to monitor development interventions through phone calls can be a relatively cheap and an effective alternative to sending SMS or to face-to-face visits, particularly in remote, low-accessibility areas.

**Table 5:** Cost-effectiveness of responses through SMS and phone calls.

Costs	SMS	Phone calls
Cost per question (US dollars)	1.12	0.85
Cost per effective response (US dollars)	7.76	1.12

## Concluding remarks

This study draws several important lessons for monitoring development interventions in rural areas through the use of cell phones.

We find a very low overall response rate to questions asked via SMS, at 14.4%, similar to that obtained during the pilot study in Chiquimula, at 12.3%. Since during the household listing we placed special emphasis on verifying the contact phone number provided by the household, the low response rate does not seem to be related to incorrect phone numbers but to a general low propensity to respond to SMS by households.

In contrast, the response rate to phone calls was over five times higher compared to SMS, at 75.8%. This points to the enormous potential of using phone calls to monitor development interventions in real time, especially in remote areas with limited accessibility. Phone calls appear to allow for more candid and personalized contact with households, diminishing households’ mistrust relative to, for example, answering SMS from an unknown phone number. Phone calls also permit to ask questions in the respondents’ native language, helping to increase trust and understanding of the information being requested from them. Similarly, phone calls allow to ask several questions in a single call, unlike SMS which cannot handle responses to multiple questions at the same time. In addition, given that incoming phone calls are free in Guatemala, this method eliminates potential liquidity constraints and avoids the need to provide airtime top-ups.

The analyses further show that language, level of education, and age of the household head are not critical barriers to obtaining answers through phone calls, while they present major obstacles when obtaining responses via SMS. Moreover, response rates obtained either by SMS or phone calls do not seem to vary according to the geographic location of a household, to whether the monitored individual is a child under two years of age or a pregnant woman, or to the type of health intervention monitored.

Furthermore, we find a high level of accuracy in the responses provided by households, with rates of agreement between household responses and administrative records of 84% and 91% for questions asked, respectively, through SMS and phone calls. This indicates, on one hand, that households are generally aware of the specific health services offered by MSPAS and, on the other hand, that they are willing to provide that information without distortion.

All in all, we conclude that monitoring development interventions using cell phones is feasible, with phone calls being superior to SMS in terms of household participation, trust, and understanding of the questions asked. In addition, the responses provided are very accurate. The lack of impact found from sending SMS reminders on the reception of health and nutrition services also seems to support the conclusion that communication via SMS is not an effective mode of contact with households.

More broadly, the almost identical SMS response rates with those previously obtained in Chiquimula, a department located in the opposite (East) side of the country with very different socioeconomic, cultural, ethnicity, and linguistic characteristics than Quiche, points towards the replicability of the results in multiple rural settings of Guatemala. Moreover, while the department of Quiche presented important challenges for the study such as low levels of education and familiarity with ICTs, multiple languages, or rugged geography and poor network connectivity, the study shows that a monitoring system based on direct phone calls to households is both feasible and effective. In this regard, we would expect our study findings to be replicable in other rural contexts around Latin America and arguably other regions around the world. Nevertheless, we recognize that assessing external validity is ultimately an empirical matter that should be further examined in future studies.

Finally, while setting up a monitoring system with calendarized phone calls requires establishing a specialized (part-time) call center, with enough teleoperators who speak all the relevant languages of the population of interest, the costs of implementing such center are lower than using a specialized automatic platform to send and record answers by SMS (in addition to providing households with airtime top-ups to respond to the SMS). If we additionally take into account the high response rate to phone calls, a monitoring system of this nature can be a low-cost, effective tool to collect timely and reliable information for program implementors, donors, and policymakers. More generally, considering the current lockdown measures and suspension of in-person field activities due to the COVID-19 crisis, phone calls can be a valuable instrument for monitoring purposes and to implement brief surveys.

## Supporting information

S1 Fig Monitoring questions by intervention. (TIF)

S2 Fig Number of monitored interventions (questions asked) by age of the child and months of pregnancy. (TIF)

S3 Fig Response rates at the household level. (TIF)

S4 Fig Response rates by intervention. The vertical lines correspond to the 95% confidence bands.
(TIF)

S1 Table Comparison between Chiquimula and Quiche studies. (DOCX)

S2 Table Household listing and monitored sample. (DOCX)

S3 Table Orthogonality test between households receiving SMS and phone calls. The table reports the corresponding averages and standard deviations in parentheses. The p-value results from the orthogonality test between the two household groups; a value larger than 0.05 indicates that the difference in each variable between the two groups is not statistically different at a 95% confidence level.
(DOCX)

S4 Table Multivariate regression analysis on the probability to respond via SMS. *** p<0.01, ** p<0.05, * p<0.1. Standard errors reported in parentheses clustered by community. Estimates include an indicator variable that takes the value of one when a household head did not report an education level or a household did not report distance to the health center, and zero otherwise. For these cases, we imputed the median distance at the community level. Model estimated by ordinary least squares.
(DOCX)

S5 Table Multivariate regression analysis on the probability to respond via mobile phone calls. Note: *** p<0.01, ** p<0.05, * p<0.1. Standard errors reported in parentheses clustered by community. Estimates include an indicator variable that takes the value of one when a household head did not report an education level or a household did not report distance to the health center, and zero otherwise. For these cases, we imputed the median distance at the community level. Model estimated by ordinary least squares.
(DOCX)

S6 Table Main reason why SMS or phone call could not be answered. Responses from 47 women that participated in the focus groups conducted.
(DOCX)

S1 Appendix Household listing and monitored sample. (DOCX)

S2 Appendix Qualitative findings. (DOCX)

S1 Data (XLS)

S1 File Dataset dictionary. (PDF)
