# Supplementary figures and images for: Assessing the use of cell phones to monitor health and nutrition interventions: Evidence from rural Guatemala
Source: PLoS One. 2020 Nov 3;15(11):e0240526. doi: 10.1371/journal.pone.0240526 (PMC7608922; doi:10.1371/journal.pone.0240526)

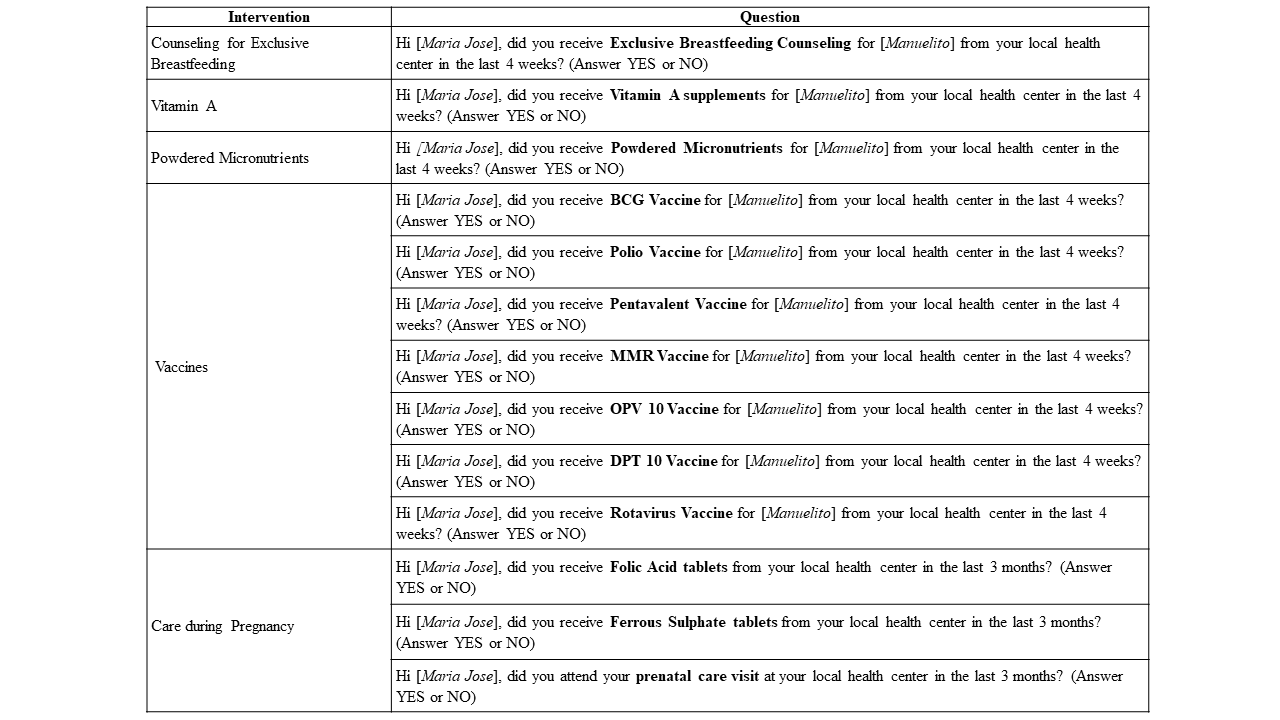

Supplement: S1 Fig — (TIF) [file pone.0240526.s001.tif]

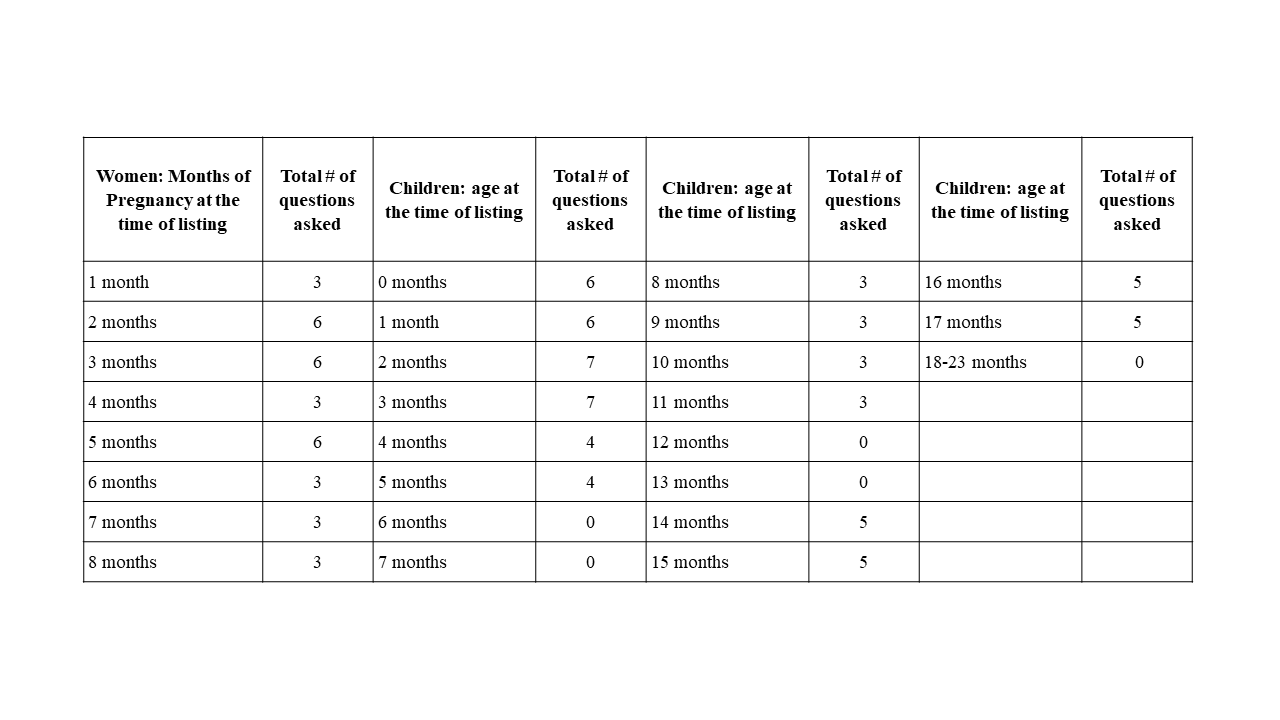

Supplement: S2 Fig — (TIF) [file pone.0240526.s002.tif]

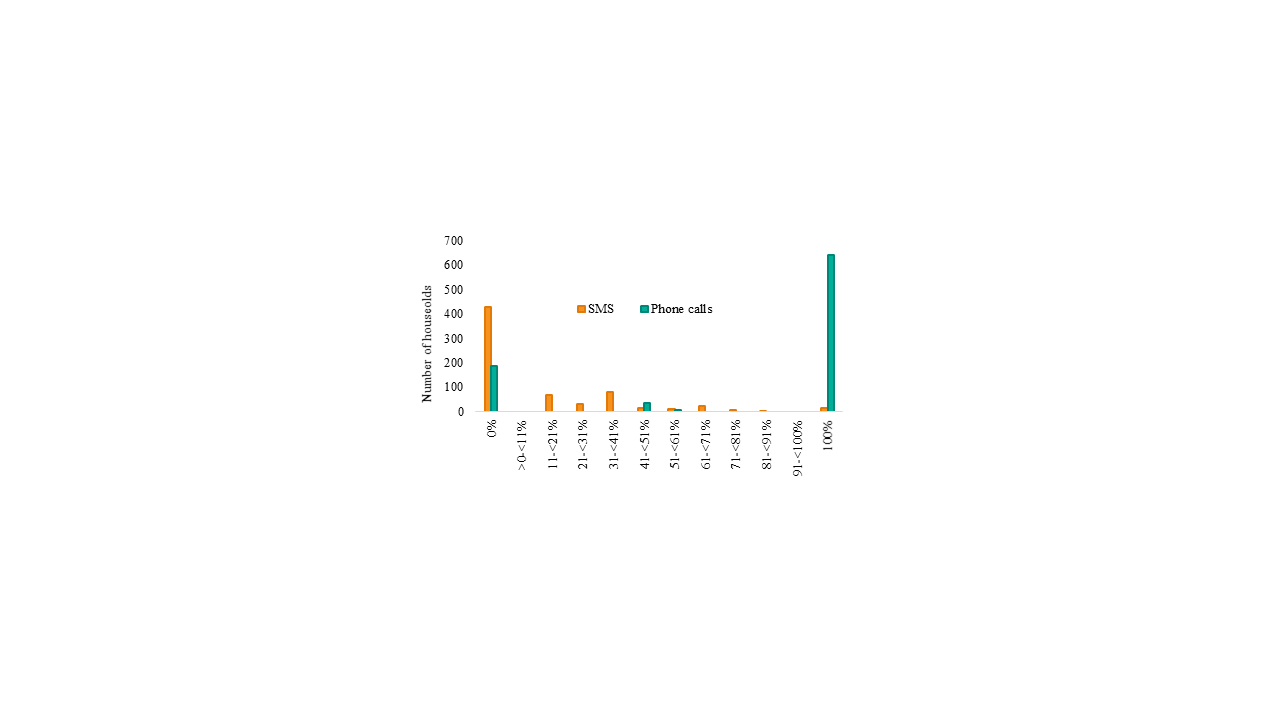

Supplement: S3 Fig — (TIF) [file pone.0240526.s003.tif]

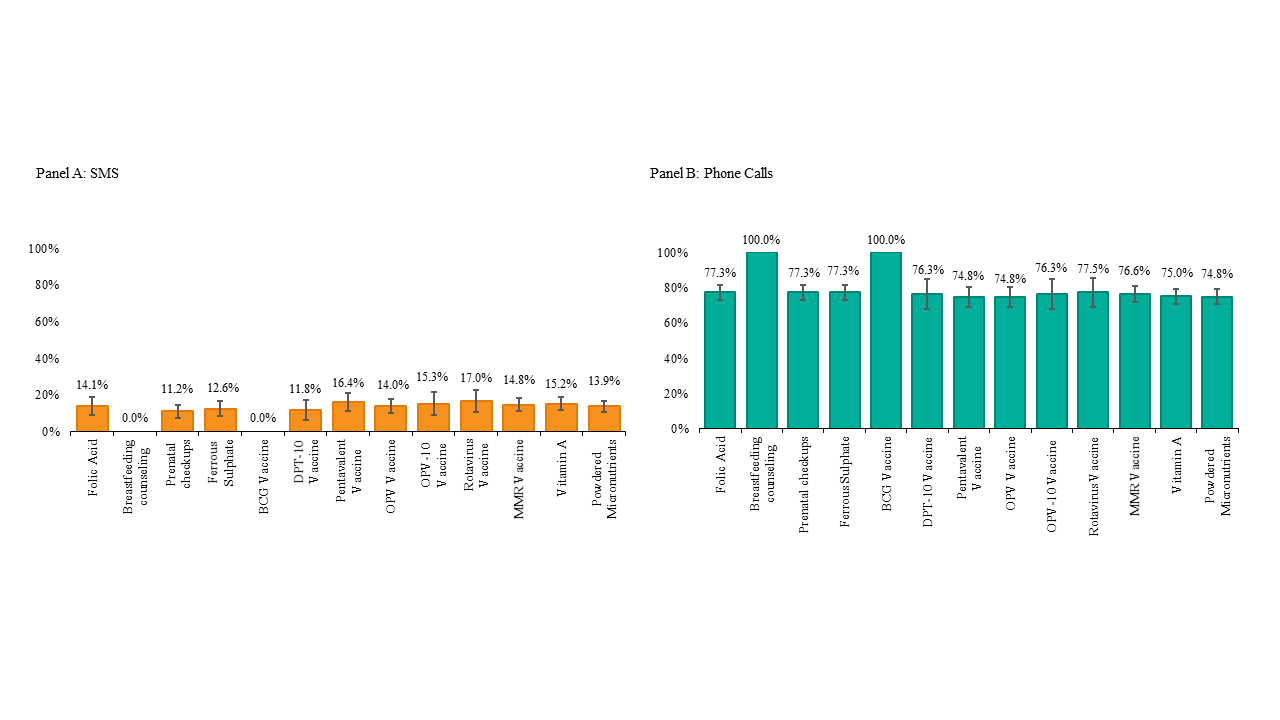

Supplement: S4 Fig — The vertical lines correspond to the 95% confidence bands. (TIF) [file pone.0240526.s004.tif]
