# Supplementary material for: Assessing the use of cell phones to monitor health and nutrition interventions: Evidence from rural Guatemala
Source: PLoS One. 2020 Nov 3;15(11):e0240526. doi: 10.1371/journal.pone.0240526 (PMC7608922; doi:10.1371/journal.pone.0240526)
Supplement: S1 Table — (DOCX) [file pone.0240526.s005.docx]

S1 Table. Comparison between Chiquimula and Quiche studies

| **Description** | **Chiquimula** | **Quiche** |
| --- | --- | --- |
| Municipalities | San Jacinto and Olopa | Nebaj and Uspantan |
| Communication days | Wednesdays and Sundays | Sundays |
| Language of communication | SMS (Spanish) | SMS (Spanish) and Phone calls (Ixil, Spanish, Kiche, and Qeqchi) |
| Conditional airtime top-ups (SMS) | Yes | No |
| Partial monitoring | Yes | No |
| Period of monitoring | 6 months | 4 months |
| Number of monitored interventions | 16 | 13 |
| Work team exclusively female | No | Yes |
| Reminders via SMS | No | Yes |
