# Supplementary material for: Assessing the use of cell phones to monitor health and nutrition interventions: Evidence from rural Guatemala
Source: PLoS One. 2020 Nov 3;15(11):e0240526. doi: 10.1371/journal.pone.0240526 (PMC7608922; doi:10.1371/journal.pone.0240526)
Supplement: S2 Table — (DOCX) [file pone.0240526.s006.docx]

S2 Table. Household listing and monitored sample

|  | **Municipality** | | **Total** |
| --- | --- | --- | --- |
|  | **Nebaj** | **Uspantan** |  |
| Number of communities | 36 | 44 | 80 |
| Number of households | 4,818 | 4,881 | 9,699 |
| Number of individuals | 23,452 | 25,552 | 49,004 |
|  |  |  |  |
| Number of households with pregnant women or children under 2 years old | 1,179 | 1,380 | 2,559 |
|  |  |  |  |
|  |  |  |  |
| Number of households fulfilling all eligibility criteria | 1,113 | 1,261 | 2,374 |
|  |  |  |  |
|  |  |  |  |
| Number of households eligible for the 4-months study | 698 | 844 | 1,542 |
|  |  |  |  |
