# Supplementary material for: Assessing the use of cell phones to monitor health and nutrition interventions: Evidence from rural Guatemala
Source: PLoS One. 2020 Nov 3;15(11):e0240526. doi: 10.1371/journal.pone.0240526 (PMC7608922; doi:10.1371/journal.pone.0240526)
Supplement: S3 Table — The table reports the corresponding averages and standard deviations in parentheses. The p-value results from the orthogonality test between the two household groups; a value larger than 0.05 indicates that the difference in each variable between the two groups is not statistically different at a 95% confidence level. (DOCX) [file pone.0240526.s007.docx]

S3 Table. Orthogonality test between households receiving SMS and phone calls

| **Characteristic** | **Households monitored via SMS** | **Households monitored via phone calls** | **p-value** |
| --- | --- | --- | --- |
| If Household Head is male | 0.875 | 0.872 | 0.849 |
|  | (0.013) | (0.011) |  |
| Household Head age | 38.539 | 39.596 | 0.125 |
|  | (0.498) | (0.467) |  |
| If Household Head speaks non-Spanish language | 0.918 | 0.856 | 0.000 |
|  | (0.011) | (0.012) |  |
| If Household Head has elementary education | 0.424 | 0.399 | 0.309 |
|  | (0.019) | (0.017) |  |
| If Household Head has secondary education | 0.208 | 0.222 | 0.489 |
|  | (0.016) | (0.014) |  |
| Number of household members | 6.171 | 6.262 | 0.513 |
|  | (0.106) | (0.091) |  |
| Distance to the health center (in minutes) | 21.327 | 20.972 | 0.793 |
|  | (0.983) | (0.916) |  |
| If pregnant woman monitored | 0.248 | 0.245 | 0.914 |
|  | (0.017) | (0.015) |  |
| If child under two years old monitored | 0.752 | 0.755 | 0.914 |
|  | (0.019) | (0.017) |  |
| Observations | 674 | 868 | 1,542 |

Note: The table reports the corresponding averages and standard deviations in parentheses. The p-value results from the orthogonality test between the two household groups; a value larger than 0.05 indicates that the difference in each variable between the two groups is not statistically different at a 95% confidence level.
