# Supplementary material for: Assessing the use of cell phones to monitor health and nutrition interventions: Evidence from rural Guatemala
Source: PLoS One. 2020 Nov 3;15(11):e0240526. doi: 10.1371/journal.pone.0240526 (PMC7608922; doi:10.1371/journal.pone.0240526)
Supplement: S5 Table — Note: *** p<0.01, ** p<0.05, * p<0.1. Standard errors reported in parentheses clustered by community. Estimates include an indicator variable that takes the value of one when a household head did not report an education level or a household did not report distance to the health center, and zero otherwise. For these cases, we imputed the median distance at the community level. Model estimated by ordinary least squares. (DOCX) [file pone.0240526.s009.docx]

**S5 Table. Multivariate regression analysis on the probability to respond via mobile phone calls**

| **Coefficients** | **(1)** | **(2)** |
| --- | --- | --- |
|  | Dependent variable: If household responded | |
| If Household Head is male | -0.033 | 0.013 |
|  | (0.037) | (0.032) |
| Household Head age | 0.002 | 0.002 |
|  | (0.002) | (0.002) |
| If Household Head speaks non-Spanish language | -0.032 | -0.055* |
|  | (0.045) | (0.032) |
| If Household Head has elementary education | 0.013 | 0.024 |
|  | (0.042) | (0.043) |
| If Household Head has secondary education | 0.014 | 0.017 |
|  | (0.038) | (0.039) |
| Number of household members | -0.005 | -0.004 |
|  | (0.006) | (0.007) |
| Distance to the health center (in minutes) | -0.001** | -0.000 |
|  | (0.001) | (0.001) |
| If monitoring care intervention during pregnancy | 0.028 | 0.004 |
|  | (0.025) | (0.025) |
| If monitoring vaccine intervention | 0.006 | 0.006 |
|  | (0.015) | (0.014) |
| If household located in Nebaj | 0.049 |  |
|  | (0.042) |  |
| Constant | 0.759*** | 0.645*** |
|  | (0.083) | (0.145) |
|  |  |  |
| Community Fixed Effects | No | Yes |
|  |  |  |
| Observations | 3,816 | 3,816 |

Note: *** p<0.01, ** p<0.05, * p<0.1. Standard errors reported in parentheses clustered by community. Estimates include an indicator variable that takes the value of one when a household head did not report an education level or a household did not report distance to the health center, and zero otherwise. For these cases, we imputed the median distance at the community level. Model estimated by ordinary least squares.
