# Supplementary material for: Assessing the use of cell phones to monitor health and nutrition interventions: Evidence from rural Guatemala
Source: PLoS One. 2020 Nov 3;15(11):e0240526. doi: 10.1371/journal.pone.0240526 (PMC7608922; doi:10.1371/journal.pone.0240526)
Supplement: S6 Table — Responses from 47 women that participated in the focus groups conducted. (DOCX) [file pone.0240526.s010.docx]

**S6 Table. Main reason why SMS or phone call could not be answered**

| **Main reason** | **SMS** | **Phone calls** |
| --- | --- | --- |
| Mistrust of my partner, he did not want me to answer | 6% | 0% |
| Lack of credit | 25% | 0% |
| Lack of access to the mobile phone (several people use the phone) | 6% | 20% |
| Lack of mobile phone signal | 13% | 0% |
| I did not know how to handle the mobile phone or answer SMS | 19% | 0% |
| I did not know they were sending SMS or phone calls, or I never received a message | 6% | 0% |
| The mobile phone owner did not notify me about the SMS or phone calls received | 25% | 40% |
| I did not understand the question | 0% | 20% |
| I lost my mobile phone | 0% | 20% |
| Total | 100% | 100% |

Note: Responses from 47 women that participated in the focus groups conducted.
