# Supplementary material for: Assessing the use of cell phones to monitor health and nutrition interventions: Evidence from rural Guatemala
Source: PLoS One. 2020 Nov 3;15(11):e0240526. doi: 10.1371/journal.pone.0240526 (PMC7608922; doi:10.1371/journal.pone.0240526)
Supplement: S1 Appendix — (DOCX) [file pone.0240526.s011.docx]

**S1 Appendix. Household listing and monitored sample**

In each of the selected communities in the municipalities of Nebaj and Uspantan, a brief household listing exercise was conducted, between November 30 and December 23, 2018, in order to identify those households with individuals of interest and obtain their georeferenced location and cell phone numbers. A short questionnaire was designed to collect the following information from each household: household size and composition, demographic information from each household member (name, gender, age, level of education, language commonly used, date of birth, and pregnancy among women), characteristics of the housing unit, name and location of the health center that household members regularly attend (as well as the time it takes to get there), home address, GPS coordinates, all home cell phone numbers, and level of familiarity with the use of text messages (SMS). Surveyors were instructed to verify the phone number provided (by calling the phone number).

Since the goal was to evaluate a monitoring system through SMS and cell phone calls, the following additional eligibility criteria were imposed:

- Access to a cell phone. The home had to have at least one cell phone number that could be reached during the duration of the study. In those cases where more than one cell phone was available, the one closest to the targeted individual (pregnant woman or the mother of the child under two years of age) was chosen. In cases where the main contact person did not have a cell phone of their own, the interviewee was asked to indicate the best contact number to locate the contact person. When the home did not have a cell phone, the cell phone of a neighbor or nearby relative was requested.
- Literacy and/or familiarity among households with the use of SMS. Given that households selected to receive questions through SMS had to be able to read, understand, and answer the questions received and that SMS communication was to be exclusively in Spanish, an additional eligibility criteria was imposed requiring at least one member of the household, older than 12 years, to be able to read and write in Spanish (the same criteria applied to the neighbor or relative, in the case of households that did not have a cell phone).

The listing covered 80 communities, 9,699 households, and 49,004 individuals in both municipalities (see Supplementary S2 Table). Of the total number of households surveyed, 2,559 (26.4%) had at least a child under two years old or one pregnant woman. Of these, 2,374 households met the aforementioned additional selection criteria. Hence, around 93% of the households with an eligible individual also had access to a cellphone and at least one literate household member, consistent with the broader cell phone penetration and household literacy rates in rural Guatemala.

Finally, since the study focused on monitoring 13 specific interventions linked to the first 1,000 days of life, it was only necessary to ask questions to women 3, 6, or 9 months pregnant and to children reaching 2, 4, 6, 12, or 18 months of age during the four monitoring months. As a result, of the total eligible individuals identified during the census and fulfilling the additional selection criteria, only ​1,542 (65%) were ultimately monitored. These individuals comprise the study sample. Note that the reduction in the number of individuals monitored with respect to those eligible (1,542 out of 2,374) is exclusively due to the relatively short duration of the monitoring period; if this monitoring system were to be scaled up and implemented continuously, all eligible individuals fulfilling the additional selection criteria would be part of the monitoring activities.
