# Supplementary material for: Assessing the use of cell phones to monitor health and nutrition interventions: Evidence from rural Guatemala
Source: PLoS One. 2020 Nov 3;15(11):e0240526. doi: 10.1371/journal.pone.0240526 (PMC7608922; doi:10.1371/journal.pone.0240526)
Supplement: S2 Appendix — (DOCX) [file pone.0240526.s012.docx]

**S2 Appendix. Qualitative Findings**

We implemented a qualitative analysis to better understand the behavior of households participating in the monitoring study via SMS and phone calls. Once the monitoring period in Quiche was over, we selected four communities to conduct focus groups: two in Nebaj (Xemamatze that received SMS and Pexla that received phone calls), and two in Uspantan (El Palmar that received SMS and Joya Larga that received phone calls). All of these locations also received SMS reminders. Roughly, 10-13 women (pregnant women or mothers of the targeted children) participated in each of the sessions.

The results of the focus groups reveal that the main reasons for not answering the questions sent by SMS were mainly related to lack of airtime (despite the airtime top-ups provided) and communication failures within the household (not being notified about the SMS as cell phones are usually shared between several family members). In the case of phone calls, the few that did not respond also mentioned miscommunication issues as they were not notified about the calls or did not have access to the phone during the whole day, as well as a lack of understanding or that they had lost their phone (see Supplementary S6 Table). As opposed to the pilot study in Chiquimula, gender issues or mistrust from the partner were not reported as relevant issues, largely because the listing (initial household visits) and the monitoring via phone calls were exclusively done by women.

Among the participants that reported answering the questions, we discussed intra-household communication and social dynamics aspects. On this matter, women reported to have discussed the SMS and phone calls with their partners, other family members, and with some neighbors, and these conversations encouraged them to answer either the SMS or phone calls received. While prior to the start of the monitoring we advertised the study through brief interviews in the local radio and television, in order to remind participating households about the study and encourage them to answer the questions they would be receiving over the following weeks, only few participants (mainly in Nebaj) reported to have heard or seen the advertisements or interviews but claimed these had stimulated them to respond.

Regarding attending health centers to receive the scheduled interventions, the participants expressed that typically there are no major reasons or factors that prevent them from regularly attending the health center when needed. This represents an additional explanation to why sending SMS reminders did not have any impact on the reception of health services, combined with the reports from participants that it was more difficult for them to engage with SMS. While someone in the household may be able to read and write in Spanish, others do not; among the participants in the focus groups, for instance, 51% reported to be illiterate.

Finally, we received suggestions for similar studies in the future. In particular, (1) it is preferable to exclusively communicate by phone calls (both for monitoring and reminders) since it is their preferred means of communication, it allows them to communicate in their local language, and it does not represent any additional costs; (2) it would help to establish a protocol on how to proceed in case they change or lose their phone number, which seems to be a recurrent pattern in rural areas in Guatemala (perhaps relying on a designated contact person in the community); and (3) it is important to count with the support of local authorities, as it enhances the credibility of the monitoring process.
