# Supplementary material for: Assessing the use of cell phones to monitor health and nutrition interventions: Evidence from rural Guatemala
Source: PLoS One. 2020 Nov 3;15(11):e0240526. doi: 10.1371/journal.pone.0240526 (PMC7608922; doi:10.1371/journal.pone.0240526)
Supplement: S1 File — (PDF) [file pone.0240526.s014.pdf]

Dataset dictionary

Variable	Definition
HHID	Household identifier
intervention	Monitored intervention (Categories 1-13)<br>1= Folic Acid<br>2= Exclusive breastfeeding<br>3= Control visit throughout pregnancy<br>4= Powdered micronutrients<br>5= Ferrous sulphate<br>6= BCG<br>7= DPT 10<br>8= Pentavalent<br>9= Polio<br>10= OPV 10<br>11= Rotavirus<br>12= MMR (Measles, Mumps, Rubella)<br>13= Vitamin A
treatment	1= Phone calls, 2= Text messages
reminders	1= Household received SMS reminder, 0= Household did not receive SMS reminder
validanswer	1= Valid answer, 0= Invalid answer
received	1= Monitored individual received the intervention, 0= Monitored individual did not receive intervention
male	1= Household head is male, 0= Household head is female
age	Household head age
nonsp_lang	1= Household head speaks non-Spanish language, 0= Household head speaks Spanish language
educ00	1= Household head has non-education, 0= Other
educ01	1= Household head has elementary education, 0= Other
educ02	1= Household head has secondary education, 0= Other
size	Number of household members

Variable	Definition
subject	Monitored individual (Categories 1-2)<br>1= Pregnant women<br>2= Child under 2 years old
vaccine_int	1= Vaccine intervention, 0= Other intervention
distance	Distance to the health center (in minutes)
mun	Municipality (Categories 1-2)<br>1= Municipality 1<br>2= Municipality 2
community	Community Identifier
distance_o	1= Distance to the health center imputed, 0= Other
educ_o	1= Household head education imputed, 0= Other
